# Examination of How Mall Visits Moderate the Impact of Adverse Weather on Daily Step Counts: A Multilevel Analysis Using Nationwide Data from a Smartphone Application

**DOI:** 10.1007/s11524-025-01031-5

**Published:** 2025-11-27

**Authors:** Hiroaki Yoshida, Yoko Matsuoka, Masamichi Hanazato

**Affiliations:** 1 Center for Preventive Medical Sciences, Chiba University, 1-33 Yayoicho, Inage-ku, Chiba-shi, Chiba, 263-8522 Japan; 2 Design Research Institute, Chiba University, Tokyo, Japan

**Keywords:** Mall, Daily step count, Climate change, Mall visits, Physical activity, Adverse weather

## Abstract

Climate change has led to an increase in extreme weather events. Adverse weather conditions limit outdoor activities, reduce step counts, and negatively affect health. Malls offer a possible alternative for walking in such weather; however, their step-loss mitigating role remains unexplored. We analyzed data from 18,666 individuals aged ≥ 18 years (mean ± standard deviation: 48.0 ± 12.6; women: 71.5%), tracking daily step counts over a year using a smartphone application. Step count data were collected via devices owned by the participants and then transferred to the program database through the application. Multilevel mixed-effects linear regression was used to estimate the associations between daily step counts and various interaction terms, focusing on two-way interactions between mall visits and weather, and three-way interactions involving sociodemographic factors. On days with maximum temperatures of < 15 °C or ≥ 35 °C, as well as during rain, deep snow depth, or strong winds, step counts were lower. However, on days when malls were visited, step counts were 1,269 higher compared with those on non-visit days. This difference was more pronounced during adverse weather: < 15 °C, ≥ 35 °C temperature, 0.1–4.9 and > 5 mm precipitation, snow depth > 3 cm, and wind speeds > 2.50 m/s. Mall visits mitigated weather-related step count reductions, particularly among older adults, women, and low-density area residents who generally take fewer steps. These findings underscore the role of weather-independent spaces, such as malls, in mitigating step count reductions during adverse weather. Improving access to and promoting their use are essential for maintaining physical activity in such conditions.

**Supplementary Information:**

The online version contains supplementary material available at 10.1007/s11524-025-01031-5.

## Introduction

Climate change is a global issue characterized by an increase in extreme weather events, such as rising temperatures and heavy rainfalls. Adverse weather conditions reduce step counts and physical activity levels. Physical activity levels in adults have been shown to be positively associated with daylight hours and negatively associated with minimum temperature, precipitation, and wind speed [1]. A study of 227 Chinese adults aged 50–70 years found an inverted U-shaped association between temperature, wind speed, and step count and a negative relationship between high relative humidity and step count [2]. Other adverse weather, such as those examined in this study (e.g., deep snow depth), may also decrease physical activity levels by making it difficult for people to step outside.

Walking is essential for health and is accessible to all able-bodied people. Higher daily step counts are associated with lower mortality from all causes, cardiovascular diseases, and cancer [3], as well as a lower risk of dementia [4] and better mental health [5]. However, lower physical activity levels have been reported in women [6], older adults, and those living in non-urban areas [7]. Understanding the factors that influence step counts and sociodemographic differences is critical for increasing individual physical activity and promoting healthy lifestyles.

Malls are noted as potential walking environments unaffected by temperature or rain [8] and as cooling shelters for heat stroke prevention [9]. These facilities are part of the urban pedestrian environment and contribute to walkability. Beyond their role as shopping destinations, malls also serve as spaces for social interaction and recreation, functioning as public spaces within the community and promoting social engagement and physical activity. However, no studies have investigated whether mall visits mitigate step count declines during adverse weathers, or how this effect varies based on sociodemographic factors. Therefore, this study aimed to determine whether mall visits can mitigate adverse weather-induced step loss, and whether the effectiveness differs by sociodemographic factors. As extreme weather events increase due to climate change, identifying environments that can prevent the decline in step counts during adverse weather is critical from both public health and urban design perspectives.

## Methods

### Study Design and Setting

**Fig. 1:**
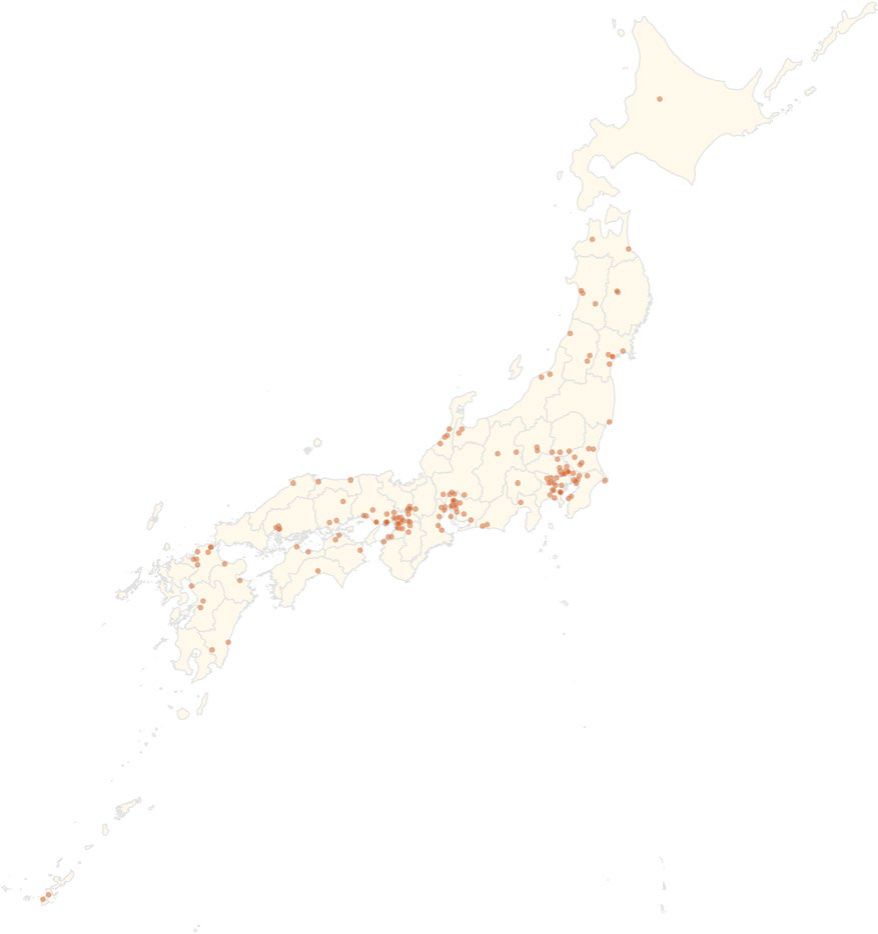
Locations of AEON MALL facilities

A cohort survey was conducted among users registered on a mall smartphone application developed by AEON MALL Co., Ltd., a nationwide mall operator in Japan that operated 164 malls as of December 2022 (Fig. 1). The company is one of the largest operators in the domestic shopping center industry, providing facilities that are easily accessible and familiar to the public. The gross leasable area of AEON MALL accounts for approximately 15% of all shopping centers in Japan, and approximately 59% of the Japanese population resides within 10 km of one of its facilities. On average, each shopping center has 50,038 m^2^ of gross leasable area. Across 163 malls in Japan, approximately 1.3 billion cumulative annual visits are recorded, which corresponds to an average of approximately 8 million visits per mall. Further details regarding AEON MALL are provided in Supplementary Table 1. The respondents were recruited through a banner on the application and invited to participate in the survey between October and December 2022. We then tracked the respondents’ mall visits and daily step count data for the following year (January to December 2023) using the application. The application, compatible with both iOS and Android, allows users to view their daily step counts and store event information.

This study used de-identified datasets and was approved by the Research Ethics Committee of the Graduate School of Medicine of Chiba University (M10050). The informed consent for research use of step count data was obtained during registration, and sociodemographic information was obtained from the survey. This study was conducted in accordance with the STROBE guidelines.

### Participants

**Fig. 2:**
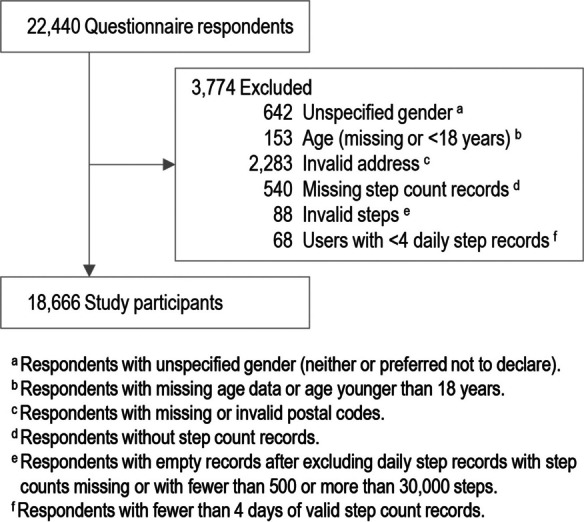
Flowchart of study participant selection

**Fig. 3:**
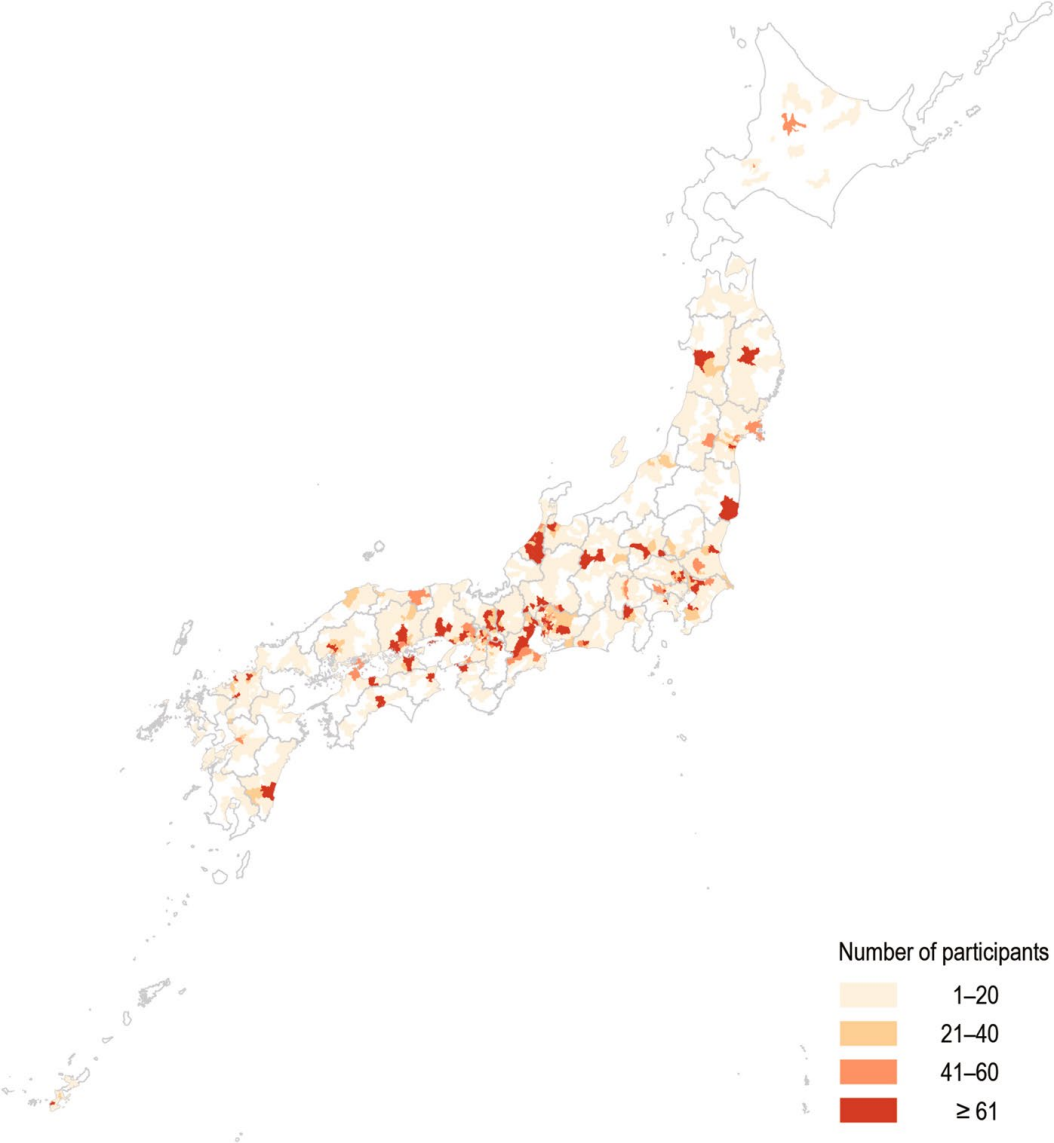
Participants' residential areas

This study analyzed data from 18,666 mall application users who were ≥ 18 years old and responded to the survey (Fig. 2). We excluded records with missing data on gender (men, women, others, or prefer not to declare) or residence and those with valid step count data of < 4 days, based on prior research that identified the minimum number of days required to reliably assess an individuals’ habitual walking behavior [10], after records with < 500 or > 30,000 steps were excluded as outliers [11]. The participants were distributed across Japan (Fig. 3).

### Outcome Variable

Daily step counts were collected from the database of the application, which were originally measured via devices owned by the participants, including iPhones, Android smartphones, and other wearable devices. In general, smartphone sensors include accelerometers and gyroscopes, although the specific sensors can vary by device. When users opened the application, the step count data for the previous 7 days were recorded in the database, regardless of mall visiting status.

### Explanatory Variable

The explanatory variable was defined as the presence or absence of mall visits, and the interactions between weather and mall visits were analyzed. Additionally, the three-way interactions involving mall visits, weather, and sociodemographic factors (gender, age group, and population density) associated with lower step counts were examined.

Tapping the ‘Start Walking’ button in the application activated GPS to confirm that the user was in the mall, only then allowing participation in the “walking program.” Mall visits days were defined as those on which the users participated in the “walking program” using the application, with the rest of the days classified as no mall visits. After completing 1,000 steps in the “walking program,” the participants became eligible to draw a lottery ticket for points that could be redeemed for shopping or gift vouchers usable at AEON Group stores. Participants who drew winning tickets received 1, 5, 10, 50, or 500 points (with 1 point approximately equivalent to US$ 0.0068), while those who drew losing tickets received no points. In addition, even without directly visiting a mall, participants were granted the right to draw a lottery if they achieved ≥ 8,000 steps per day on five separate days. At the time of this survey (December 2022), the walking program had 584,762 registered users, including inactive ones. By 2023, this walking program was available in 144 malls. Further details of the “walking program” are provided in our previous work [12].

Weather data were obtained from the Agro-Meteorological Grid Square Data operated by the National Agriculture and Food Research Organization. This system provides meteorological data at a resolution of approximately 1 km^2^. Based on a systematic review of weather and physical activity suggesting negative associations between physical activity and certain weathers [13], we focused on temperature, precipitation, wind speed, and snow depth. In this study, we obtained daily precipitation, snow depth, average wind speed, and maximum temperature measurements. Weather data were averaged using a geographic information system (GIS) based on the meteorological information for residential areas identified by postal codes from the questionnaire. ArcGIS Pro 3.1.0 (Esri, Redlands, CA, USA) was used for GIS analysis.

Hot days were defined as days with maximum temperatures of ≥ 35 °C, based on the Japan Meteorological Agency’s definition of extremely hot days; comfortable days had a maximum temperature of 15.1–34.9 °C; ≤ 15 °C was used to define cold days based on studies associating lower maximum temperatures with low physical activity [1, 14]. Hot and cold days are associated with reduced physical activity [13]. Days were further stratified based on precipitation (0, 0.1–4.9, or ≥ 5.0 mm), snow depth (0, 0.1–2.9, or ≥ 3.0 cm), and wind speed (< 1.74, 1.74–2.50, or > 2.50 m/s).

### Covariates

Sociodemographic status, health behaviors, and health status were assessed using a self-report questionnaire. The data collected as sociodemographic status included gender, age group (18–39, 40–64, or ≥ 65 years), marital status (unmarried, with a spouse, widowed or divorced, or other), employment status (employed, employed as a part-time worker, on leave of absence, or not employed), years of education (< 12, 12–13, or ≥ 14 years), body mass index (underweight/normal weight < 24.9, overweight/obese ≥ 25.0 kg/m^2^, or invalid), and driving status (non-drivers: no driving; or drivers: ≥ 1–3 days/month). Annual equivalent income was calculated by dividing household income by the square root of household size and categorized into < 2.0, 2.0–3.9, or ≥ 4.0 million yen. Population density, calculated based on postal codes in the questionnaire, was divided into tertiles (< 3,208, 3,208–7,265, or > 7,265 persons/km^2^). We collected data on self-rated health (excellent, very good, good, fair, poor, or very poor) and divided them into two categories ([excellent, very good, or good] or [fair, poor, or very poor]). Using the Transtheoretical Model of Behavior Change to evaluate health behaviors [15], participants classified their walking behavior into five stages. In the questionnaire, “walking” was defined as bouts lasting ≥ 10 min (including walking while shopping, commuting, during work breaks, or strolling), and “regularly” as ≥ 2 times per week for ≥ 20 min per session. Accordingly, the stages were: pre-contemplation (“I do not engage in walking and have no intention to start”); contemplation (“I do not engage in walking but plan to within 6 months”); preparation (“I engage in walking but not regularly”); action (“I engage in walking regularly for less than 6 months”); and maintenance (“I engage in walking regularly for 6 months or more”). The day of the week was categorized as weekday or weekend based on step count data from the mall application. These variables have been associated with daily step counts [7, 16, 17] and physical activity [18].

### Statistical Analysis

To address the potential multicollinearity among weather variables, Spearman’s correlation was used to confirm that no strong correlations exist between precipitation, snow depth, wind speed, and maximum temperature (Supplementary Table 1). A multilevel analysis with a linear mixed-effects model was conducted to estimate the regression coefficients between the explanatory variables and daily step count. The estimation was performed using iterative generalized least squares. First, we tested a null model that included only random effects for the day (level 1), individual (level 2), and region (level 3) with a fixed intercept to calculate the intraclass correlation coefficient. Furthermore, we examined a random slope model that included mall visits and the interaction between mall visits and the weather as fixed effects. Additionally, we examined the association between the daily step count and three-way interactions including mall visits, weather and sociodemographic factors. Furthermore, as a supplementary analysis, we examined the association between adverse weather and mall visits themselves. In this model, the outcome variable was mall visits (yes/no), and the explanatory variables were weather conditions defined as described above. A multilevel modified Poisson regression analysis was applied, adjusting for the same covariates as those in the main analysis. Statistical significance was set as a two-tailed p-value of < 0.05. Statistical analyses were conducted using MLwiN, version 3.06 (Centre for Multilevel Modeling, UK), and STATA, version 18.0 (StataCorp LLC, USA), with the runmlwin command.

## Results

The mean (± standard deviation) step count during the study period was 6,382 ± 4,586 for women and 9,121 ± 5,583 for men. Descriptive statistics are presented in Table 1. Women accounted for 71.5% of the participants. The 40–64 and the ≥ 65 years old groups were the largest (64%) and smallest (9.8%), respectively. Maximum temperatures were ≤ 15 °C on 26.5% of the days, 15.1–34.9 °C on 68.4% (most common), and ≥ 35 °C on 5.1%. Rainfall was absent on 67% of the days, and snow depth was 0 cm on 96.3%. Mall visits occurred on 34.5% of the days.

**Table 1:** Descriptive statistics of individual-, community-, and day-level variables

	n	Percentage
*Individual-level variables (n* = *18,666)*		
Gender		
Men	5,311	28.5
Women	13,355	71.5
Age groups (years)		
18–39	4,892	26.2
40–64	11,942	64.0
≥ 65	1,832	9.8
Body mass index (kg/m^2^)		
< 25	14,420	77.3
≥ 25	4,139	22.2
Missing	107	0.6
Marital status		
Unmarried	3,638	19.5
Spouse (including common-law marriage)	13,683	73.3
Widowed or divorced	1,263	6.8
Other	82	0.4
Employment status		
Employed	7,724	41.4
Employed as a part-time worker	5,715	30.6
On leave of absence	574	3.1
Not employed	4,653	24.9
Education		
< 12	5,369	28.8
12–13	5,884	31.5
≥ 14	7,186	38.5
Unknown and No response	227	1.2
Annual equivalent income (in millions of yen^a^)		
Tertile 1 (low: < 2.0)	5,922	31.7
Tertile 2 (mid: 2.0–3.9)	6,304	33.8
Tertile 3 (high: > 3.9)	6,440	34.5
Driving status		
Drivers	14,478	77.6
Non-drivers	4,188	22.4
Walking behavior		
Pre-contemplation	1,476	7.9
Contemplation	2,256	12.1
Preparation	6,130	32.8
Action	1,380	7.4
Maintenance	7,424	39.8
Self-rated health		
Fair poor or very poor	4,813	25.8
Good very good excellent	13,853	74.2
*Community-level variables (n* = *9,877)*		
Population density (persons/km^2^)		
Tertile 1 (low: < 3,208)	3,292	33.3
Tertile 2 (mid: 3,208–7,265)	3,293	33.3
Tertile 3 (high: > 7,265)	3,292	33.3
*Day-level variables (n* = *5,040,079)*		
Maximum temperature (°C)		
≤ 15.0	1,336,129	26.5
15.1–34.9	3,445,886	68.4
≥ 35.0	258,064	5.1
Precipitation (mm)		
0	3,378,353	67.0
0.1–4.9	828,159	16.4
≥ 5.0	833,567	16.5
Snow depth (cm)		
0	4,852,892	96.3
0.1–2.9	97,762	1.9
≥ 3.0	89,425	1.8
Wind speed (m/s)		
< 1.74	1,665,138	33.0
1.74–2.50	1,668,271	33.1
> 2.5	1,706,670	33.9
Day of the week		
Weekdays	3,595,308	71.3
Weekends	1,444,771	28.7
Mall visits		
No	3,301,928	65.5
Yes	1,738,151	34.5

^a^1 JPY = 0.007 USD (average value in November 2022)

Table 2 presents the multilevel analysis results for two-way interactions and step counts. Compared with comfortable days, step counts were 158 lower (95% confidence interval [CI]: − 168 to − 149) on cold days and 395 lower (95% CI: − 413 to − 378) on hot days. For precipitation, compared with 0 mm, step counts were 226 lower (95% CI: − 236 to − 216) for 0.1–4.9 mm and 587 lower (95% CI: − 597 to − 576) for ≥ 5 mm. For snow depth, compared with 0 cm, step counts were 122 lower (95% CI: − 149 to − 95) for 0.1–2.9 cm and 73 lower (95% CI: − 103 to − 43) for ≥ 3 cm. For wind speed, compared with < 1.74 m/s, step counts were 19 lower (95% CI: − 28 to − 9) for 1.74–2.50 m/s and 29 lower (95% CI: − 39 to − 18) for ≥ 2.50 m/s.

**Table 2:** Mixed-effect linear regression model coefficients showing an association between mall visits, weather, two-way interaction terms, and daily steps walked

Daily steps	Coefficient (95% CI)
Maximum temperature (°C)	
≤15.0	**−158 (−168 to −149)**
15.1–34.9	ref
≥35.0	**−395 (−413 to −378)**
Precipitation (mm)	
0	ref
0.1–4.9	**−226 (−236 to −216)**
≥5.0	**−587 (−597 to −576)**
Snow depth (cm)	
0	ref
0.1–2.9	**−122 (−149 to −95)**
≥3.0	**−73 (−103 to −43)**
Wind speed (m/s)	
<1.74	ref
1.74–2.50	**−19 (−28 to −9)**
>2.50	**−29 (−39 to −18)**
Mall visit	
No	ref
Yes	**1268 (1253 to 1282)**
Mall visit × Maximum temperature (≤15.0 °C)	**94 (79 to 110)**
Mall visit × Maximum temperature (≥35.0 °C)	**37 (8 to 66)**
Mall visit × Precipitation (0.1–4.9 mm)	**18 (0 to 35)**
Mall visit × Precipitation (≥5.0 mm)	**83 (65 to 101)**
Mall visit × Snow depth (0.1–2.9 cm)	47 (−1 to 96)
Mall visit × Snow depth (≥3.0 cm)	**156 (104 to 209)**
Mall visit × Wind speed (1.74–2.50 m/s)	7 (−9 to 23)
Mall visit × Wind speed (>2.50 m/s)	**19 (2 to 35)**
Gender	
Men	ref
Women	**−2256 (−2382 to −2131)**
Age groups (years)	
18–39	ref
40–64	**269 (147 to 391)**
≥65	−117 (−325 to 91)
Body mass index	
<25	ref
≥25	**−325 (−441 to −210)**
Invalid	−594 (−1222 to 34)
Marital status	
Unmarried	ref
Spouse (including common-law marriage)	**−588 (−724 to −452)**
Widowed or divorced	**−320 (−543 to −98)**
Other	−275 (−1000 to 449)
Employment status	
Employed	ref
Employed as a part-time worker	−6 (−133 to 121)
On leave of absence	**−856 (−1141 to −570)**
Not employed	**−535 (−676 to −394)**
Education	
<12	ref
12–13	**−216 (−339 to −93)**
≥14	−73 (−197 to 51)
Unknown and No response	72 (−369 to 513)
Annual equivalent income (in millions of yen^a^)	
Tertile 1 (low: <2.0)	ref
Tertile 2 (mid: 2.0–3.9)	**−125 (−246 to −4)**
Tertile 3 (high: >3.9)	**−202 (−330 to −75)**
Driving status	
Drivers	ref
Non-drivers	**730 (605 to 855)**
Walking behavior	
Pre-contemplation	ref
Contemplation	18 (−198 to 234)
Preparation	**473 (285 to 661)**
Action	**1355 (1112 to 1598)**
Maintenance	**2696 (2507 to 2884)**
Self-rated health	
Fair, poor or very poor	ref
Good, very good excellent	**263 (153 to 374)**
Day of the week	
Weekdays	ref
Weekends	**−86 (−93 to −80)**
Population density (persons per square km of inhabitable area)	
Tertile 1 (low: <3,208)	ref
Tertile 2 (mid: 3,208–7,265)	86 (−42 to 215)
Tertile 3 (high: >7,265)	**446 (314 to 579)**
Constant	7281 (7019 to 7543)

*CI* confidence interval, *ref* reference group. Bold text indicates *p*<0.05. ^a^1 JPY = 0.007 USD (average value in November 2022)

The intraclass correlation coefficient was 0.50 at the individual level and 0.04 at the regional level, indicating that individual variance accounted for half the total variance

Compared with non-visit days, mall visit days were associated with 1,268 more steps (95% CI: 1,253 to 1,282). This difference was more pronounced under adverse weather conditions. Compared with comfortable days, step counts were 94 higher (95% CI: 79 to 110) on cold days and 37 higher (95% CI: 8 to 66) on hot days. Compared with days with 0 mm of precipitation, step counts were 18 higher (95% CI: 0 to 35) on days with 0.1–4.9 mm of precipitation and 83 higher (95% CI: 65 to 101) on days with ≥ 5 mm of precipitation. Compared with days with 0 cm of snow depth, step counts were 156 higher (95% CI: 104 to 209) on days with ≥ 3 cm of snow. Finally, compared with days with wind speeds below 1.74 m/s, step counts were 19 higher (95% CI: 2 to 35) on days with ≥ 2.50 m/s of wind.

**Fig. 4:**
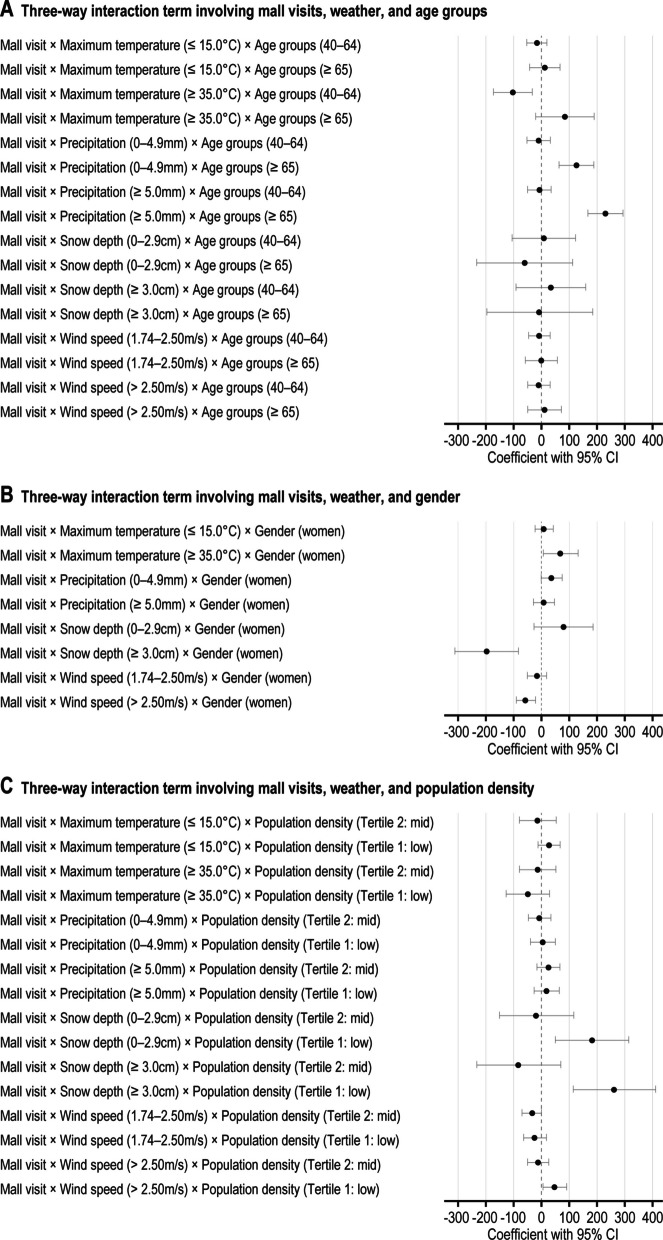
Mixed-effect linear regression model coefficients showing an association of the three-way interaction term involving mall visits, weather, and sociodemographic factors (age groups (**A**), gender (**B**), and population density (**C**)), with daily step counts. Adjusted for gender, age groups, body mass index, marital status, employment status, education, annual equivalent income, car use, walking behaviors, self-rated health, day of the week, population density, and mall visits. The three-way interaction terms involving mall visits, weather, and sociodemographic factors were separately included in the model. The reference group for this analysis includes mall visits (non-visits), maximum temperature (15.1–34.9 °C), precipitation (0 mm), snow depth (0 cm), wind speed (< 1.74 m/s), age groups (< 40 years), gender (men), and population density (tertile 3: high)

Figure 4 and Supplementary Table 3a–c show multilevel analysis results for three-way interactions and step counts. Compared with those aged < 40 years, those aged ≥ 65 years showed greater differences in step counts associated with mall visits on rainy days: 128 higher (95% CI: 65 to 191) for 0–4.9 mm of rainfall and 233 higher (95% CI: 169 to 296) for ≥ 5 mm of rainfall. Women showed greater differences in step counts associated with mall visits than did men. This difference was 68 higher (95% CI: 5 to 131) on hot days compared with those on comfortable days. However, the difference was smaller on days with ≥ 3.0 cm of snow, with step counts 198 lower (95% CI: − 312 to − 83), and on days with high wind speeds, step counts were 56 lower (95% CI: − 92 to − 20), compared with days with 0 cm snow depth and low wind speeds. Residents in low-density areas showed greater differences in steps associated with mall visits on days with snow depths of 0.1–2.9 cm and ≥ 3.0 cm compared with those in high-density areas, with step counts 182 higher (95% CI: 51 to 314) and 263 higher (95% CI: 114 to 412), respectively. Additionally, residents in low-density areas showed greater differences in steps associated with mall visits on days with wind speeds of ≥ 2.5 m/s, compared with those in high-density areas, with step counts 48 higher (95% CI: 6 to 90).

In the supplementary analysis examining the association between adverse weather and mall visits (Supplementary Table 4), colder, hotter, and windier days were more likely to be associated with mall visits, and days with precipitation or snow were less likely to be associated with mall visits.

## Discussion

The higher number of steps associated with mall visits exceeded the lower steps taken under all weather conditions. Mall visits may contribute to increased steps by encouraging movement within the mall, traveling to the mall, and stopping at destinations outside the mall. This finding is consistent with that of previous research [12]. In this study, even after adjusting for various sociodemographic factors, health conditions, and weather effects, mall visits were associated with significantly higher step count.

In adverse weather conditions, such as cold or hot temperatures, precipitation, deep snow, or strong winds, the number of steps associated with mall visits was even greater than those in comfortable weather conditions. Residents in highly walkable areas showed smaller declines in step count during summer than residents in low-walkability areas [19]. In highly walkable areas, the abundance of nearby destinations, such as grocery stores, restaurants, and public transportation, was associated with a higher likelihood of walking to these locations [20]. Even in adverse weather, using these stores and services may help mitigate step count reductions. This study found that visits to malls helped mitigate the decline in step count due to adverse weather, regardless of population density, which is a key component of walkability. Malls provide an environment in which people can walk regardless of the weather, besides offering access to a variety of services, including food, household goods, clothing, entertainment, and cultural activities. An environment that is conducive to browsing and window shopping has a potential to encourage increased step counts associated with mall visits on adverse weather days.

On days with deep snow, the higher step counts associated with mall visits exceeded the lower step counts owing to snow depth. Snow depth, more than other adverse weather conditions, significantly restricts outdoor activities and may limit activity outside the mall but increasing the time spent inside. Consequently, walking opportunities inside the mall may have increased, resulting in an overall higher step counts, exceeding the reduction typically caused by snow depth. Living in areas with snow-preventing arcades in front of homes may help maintain or even increase physical activity during snowy seasons [21]. Such arcades create an environment that supports access to destinations. This study suggests that destination environments can help mitigate step count reductions during adverse weather.

Compared with older adults, those aged < 40 years had higher step counts associated with mall visits (Supplementary Table 3a). Younger individuals are likely to engage in more shopping and window-shopping activities, resulting in higher step counts associated with mall visits. However, on rainy days, older adults showed higher step counts associated with mall visits than those aged < 40 years (Fig. 4; Supplementary Table 3a). Older adults are more affected by adverse weather conditions, such as high temperatures and wet-bulb temperature indices, resulting in lower step counts than for younger individuals [22]. This study also found that older adults had significantly lower step counts not only on hot days but also on rainy and windy days.

Owing to their heightened sensitivity to adverse weather such as rainy days [23], older adults may reduce outdoor activities before and after mall visits, resulting in longer stays in the mall. The walkable environment of a mall, together with the prolonged stay, may encourage walking, which may explain the significantly higher step counts associated with mall visits among older adults on rainy days. Outdoor activities are often restricted on hot or snowy days, leading to longer stays in malls and higher step counts in all age groups. In contrast, strong winds, which have been reported to have only limited association with physical activity [13], are not likely to limit outdoor activities across all age groups. As such, it appears that no significant differences in the step counts associated with mall visits were observed between the age groups during these adverse weather conditions (hot days, snowy days, and windy days).

Women showed higher step counts associated with mall visits (Supplementary Table 3b). Women tend to avoid unsafe or poorly maintained areas [24] and often spend more time shopping [25]. These factors suggest that the safe and well-managed environment of malls may contribute to the observed higher step counts among women.

On hot days, the higher step counts associated with mall visits was more pronounced for women (Fig. 4; Supplementary Table 3b). Women are more likely to avoid UV exposure [26] and may limit outdoor activities on hot days, preferring to spend more time in cool and comfortable environments such as malls. This is likely to have further increased the number of steps taken by women. However, the step counts associated with mall visits were lower among women on days with significant snow depth or strong winds. Significant snow accumulation likely imposes greater restrictions on activities [27], leading not only women, who tend to be more affected by the weather [28], but also men to reduce outdoor activities before and after mall visits, thereby resulting in longer mall visits. Additionally, on days with significant snow depth, outdoor activities, such as snow removal, which men often perform, are likely to increase. Consequently, the step counts associated with mall visits on days with heavy snow accumulation were higher for men. Wind speed is likely to have a lesser impact on behavior than other adverse weather conditions [13], making it more likely that both men and women will maintain activity patterns similar to those under normal conditions. Men, who tend to engage in a wider range of activities and visit more destinations [29] may experience a particularly notable increase in steps when the impact of adverse weather is minimal. These higher step counts are likely to reflect not only activities within the mall but also the additional influence of activities before and after the visit.

No significant interaction was observed between population density and mall visits (Supplementary Table 3c). However, significant results were obtained for three-way interaction terms that included weather factors (Fig. 4; Supplementary Table 3c). Residents in low-density areas exhibited higher step counts associated with mall visits on snowy days. In non-urban low-density areas, grocery stores and public transportation are not easily accessible [30]. Consequently, on snowy days when outdoor activities are severely limited, residents in low-density areas are more likely to avoid going outside. However, malls, which provide a safe and convenient indoor walking environment, may contribute to the higher step counts during visits. Similarly, residents in low-density areas showed higher step counts associated with mall visits on windy days. In such areas, private car use is common, and wind has less impact on mobility. In contrast, in high-density areas where walking and public transportation are more common, high winds may discourage people from traveling. Consequently, residents of low-density areas are likely to maintain their activities before and after the mall visit on the same day, which appears to be associated with the notably higher step counts during these visits. For people who tend to take fewer steps, such as older adults, women, and residents of low-density areas, malls provide an environment in which they can walk safely and comfortably with minimal exposure to adverse weather conditions, thereby increasing step counts.

Furthermore, regarding the association between mall visits and adverse weather, the probability of visiting malls was higher on cold days, hot days, and windy days (Supplement Table 4). On such days with extreme temperatures or strong winds, shopping malls may have been chosen as an alternative to outdoor activities. In contrast, the probability of mall visits was lower on rainy or snowy days (Supplement Table 5), suggesting that the potential benefits of malls in maintaining step counts under these conditions may not have been fully realized. These findings imply the need to strengthen both the motivations (e.g., issuing shopping coupons) and means (e.g., increasing bus services operated by malls, introducing or promoting new mobility options such as on-demand transportation) that facilitate access to malls during adverse weather.

According to previous research, cooling centers are defined as publicly accessible indoor spaces that provide relief during periods of extreme heat, including public facilities such as community halls, civic centers, and libraries, as well as shopping malls [31, 32]. This study demonstrated that shopping malls may help mitigate reductions in step counts on adverse weather days, highlighting their potential to function not only as places of refuge during extreme heat events but also as venues that support the maintenance of physical activity. Future studies should examine whether other facilities functioning as cooling centers could similarly mitigate reductions in step counts during adverse weather. Accumulating such evidence will be important for exploring urban design strategies that enhance resilience to adverse weather and extreme heat in the context of climate change.

The strengths of our study include the use of nationwide data for analysis. Additionally, we surveyed the participants to collect sociodemographic characteristics, health behaviors, and health status information, enabling us to account for various factors that influence step count. This comprehensive approach enhances the reliability of our findings.

This study has some limitations. First, variations in device types and wearing positions for measuring step counts may have caused measurement errors. However, a systematic review found that smartphone apps and wearables generally have high accuracy, suggesting minimal measurement error [33]. Second, we cannot completely remove the influence of incentives from the walking program on step counts during mall visits. As described earlier, participants could obtain lottery opportunities for points redeemable at affiliated stores. Given their low monetary value, restricted usability, and the fact that they were not exclusive to mall visits, the incentives may have had only a modest additional effect on encouraging mall visits or increasing step counts. Nevertheless, we cannot exclude the possibility that they influenced participants’ walking behavior. Third, the distinction between mall and non-mall visits may not be accurately reflected in step count data. For example, if the participants forgot to press the “Start Walking” button during a mall visit, days with significant mall-related walking would have been classified as non-mall visit days, inflating step counts on non-mall visit days. Conversely, participants may not have activated the application on brief mall visits, potentially leading to an overestimation of step counts on mall-visit days. Additionally, although records with fewer than 500 daily steps were excluded, non-mall visit days could include days participants stayed home, underestimating their step counts. These potential misclassifications could have biased the observed differences in step counts between mall and non-mall visits in either direction. Lastly, the participants were residents of Japan, limiting the generalizability of the findings to other racial or ethnic groups and developing countries.

## Conclusion

With the increasing frequency of extreme weather conditions caused by climate change, such as hot days and heavy rainfalls, addressing the reduction in physical activity and disparities in step counts caused by adverse weather is increasingly crucial for public health. Our findings highlight the importance of weather-independent environments in maintaining walking behavior. Developing weather-independent environments and enhancing accessibility to such spaces may encourage their use, promote walking, and contribute to reducing health disparities.

## Supplementary Information

Below is the link to the electronic supplementary material.

ESM 1 (DOCX 327 KB)

## Data Availability

The datasets used in this study are owned by a private company and therefore are not publicly available due to privacy considerations of smartphone application users. However, these datasets can be provided by the corresponding author upon reasonable request.
