# Supplementary material for: Examination of How Mall Visits Moderate the Impact of Adverse Weather on Daily Step Counts: A Multilevel Analysis Using Nationwide Data from a Smartphone Application
Source: J Urban Health. 2025 Nov 27;102(6):1252–65. doi: 10.1007/s11524-025-01031-5 (PMC12738399; doi:10.1007/s11524-025-01031-5)
Supplement: Supplementary file 1 — (DOCX 327 KB) [file 11524_2025_1031_MOESM1_ESM.docx]

**Additional file 1**

| Supplementary Table 1. Overview of AEON MALL Facilities in Japan | | | |
| --- | --- | --- | --- |
| No | Item | Calculation method | Source |
| 1 | Gross leasable area of AEON MALL | Summed the gross leasable area of all domestic AEON MALL facilities reported in the Data Pages. Total: 8,206,300 m². | AEON MALL Co., Ltd. Financial Results Presentation 2024. Accessed August 26, 2025  https://www.aeonmall.com/ir/pdf/ircalendar/01.pdf |
| 2 | Gross store area of shopping centers in Japan | Gross store area of 3,037 shopping centers nationwide: 54,106,743 m². | Japan Council of Shopping Centers. Shopping Center White Paper 2025 (SC Hakusho 2025). Accessed August 26, 2025.  <https://www.jcsc.or.jp/sc_data/data/overview> |
| 3 | Proportion of AEON MALL in Japan’s total shopping center floor area | Calculated as AEON MALL’s gross leasable area (8,206,300 m²) divided by the gross store area of shopping centers in Japan (54,106,743 m²), yielding 15.17%. | Derived from items 1 and 2. |
| 4 | Population within a 10-km radius (buffer) of AEON MALL facilities | Created 10-km buffers around each facility and aggregated population counts using GIS using the Census 2020 basic unit block data. A total of 74,221,004 residents (58.84% of the national population of 126,146,099) lived within 10 km of an AEON MALL. | Statistics Bureau, Ministry of Internal Affairs and Communications, Japan. Population Census 2020, Basic Unit Block Data. |
| 5 | Mean leasable area of a single AEON MALL | Calculated as AEON MALL’s total gross leasable area (8,206,300 m²) divided by the number of malls (164), yielding 50,038.41 m². | AEON MALL Co., Ltd. Financial Results Presentation 2024. Accessed August 26, 2025  https://www.aeonmall.com/ir/pdf/ircalendar/01.pdf |
| 6 | Annual cumulative number of visitors to AEON MALL facilities | The total annual number of visits to 163 AEON MALLs in Japan was approximately 1.3 billion. This cumulative value was then divided by the number of malls (163) to yield a mean of about 8 million annual visits per mall. | AEON MALL Co., Ltd. Mall Media Guide 2023. Accessed August 26, 2025. <https://www.aeon-mall.jp/mallmedia2023/> |
| 7 | Interior photograph of AEON MALL | 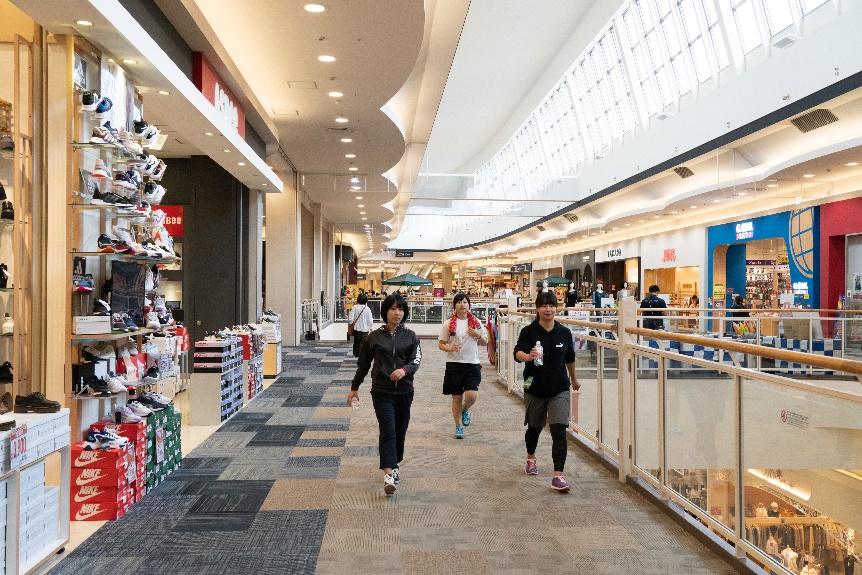  Note: This photograph was taken at AEON MALL Miyazaki. | |

Supplementary Table 2. Spearman’s rank correlation coefficients between weather variables.

|  | Precipitation | Snow depth | Wind speed |
| --- | --- | --- | --- |
| Precipitation |  |  |  |
| Snow depth | 0.05 |  |  |
| Wind speed | 0.03 | 0.04 |  |
| Maximum temperature | 0.03 | −0.29 | −0.02 |

Supplementary Table 3a. Mixed-effect linear regression model coefficients showing an association with the three-way interaction term involving mall visits, weather and age groups, and daily step counts.

| Daily steps | | Coefficient (95% CI) |
| --- | --- | --- |
| Mall visit | |  |
|  | No | ref |
|  | Yes | **1274 (1244 to 1303)** |
| Maximum temperature (°C) | |  |
|  | ≤15.0 | **−165 (−183 to −147)** |
|  | 15.1–34.9 | ref |
|  | ≥35.0 | **−319 (−353 to −284)** |
| Precipitation (mm) | |  |
|  | 0 | ref |
|  | 0–4.9 | **-179 (-199 to -159)** |
|  | ≥5.0 | **-444 (-465 to -424)** |
| Snow depth (cm) | |  |
|  | 0 | ref |
|  | 0–2.9 | **−129 (−183 to −75)** |
|  | ≥3.0 | **−101 (−160 to −42)** |
| Wind speed (m/s) | |  |
|  | <1.74 | ref |
|  | 1.74–2.50 | **−21 (−40 to −2)** |
|  | >2.50 | **−27 (−47 to −6)** |
| Age groups (years) | |  |
|  | 18–39 | ref |
|  | 40–64 | **298 (174 to 421)** |
|  | ≥65 | 73 (−137 to 283) |
| Mall visit × Maximum temperature (≤15.0°C) | | **99 (68 to 131)** |
| Mall visit × Maximum temperature (≥35.0°C) | | **109 (49 to 170)** |
| Mall visit × Precipitation (0–4.9 mm) | | 15 (−22 to 51) |
| Mall visit × Precipitation (≥5.0 mm) | | **79 (43 to 116)** |
| Mall visit × Snow depth (0–2.9 cm) | | 48 (−49 to 146) |
| Mall visit × Snow depth (≥3.0 cm) | | **138 (30 to 247)** |
| Mall visit × Wind speed (1.74–2.50 m/s) | | 12 (−21 to 45) |
| Mall visit × Wind speed (>2.50 m/s) | | 25 (−9 to 59) |
| Maximum temperature (≤15.0°C) × Age groups (40–64) | | 4 (−17 to 25) |
| Maximum temperature (≤15.0°C) × Age groups (≥65) | | **44 (9 to 80)** |
| Maximum temperature (≥35.0°C) × Age groups (40–64) | | **−64 (−104 to −23)** |
| Maximum temperature (≥35.0°C) × Age groups (≥65) | | **−395 (−463 to −327)** |
| Precipitation (0–4.9 mm) × Age groups (40–64) | | **−45 (−68 to −21)** |
| Precipitation (0–4.9 mm) × Age groups (≥65) | | **−203 (−243 to −162)** |
| Precipitation (≥5.0 mm) × Age groups (40–64) | | **−143 (−167 to −119)** |
| Precipitation (≥5.0 mm) × Age groups (≥65) | | **−553 (−593 to −513)** |
| Snow depth (0–2.9 cm) × Age groups (40–64) | | 7 (−57 to 70) |
| Snow depth (0–2.9 cm) × Age groups (≥65) | | 25 (−82 to 131) |
| Snow depth (≥3.0 cm) × Age groups (40–64) | | 53 (−17 to 123) |
| Snow depth (≥3.0 cm) × Age groups (≥65) | | −79 (−196 to 38) |
| Wind speed (1.74–2.50 m/s) × Age groups (40–64) | | 5 (−18 to 27) |
| Wind speed (1.74–2.50 m/s) × Age groups (≥65) | | −7 (−44 to 31) |
| Wind speed (>2.50 m/s) × Age groups (40–64) | | 4 (−21 to 28) |
| Wind speed (>2.50 m/s) × Age groups (≥65) | | **−51 (−91 to −10)** |
| Mall visit × Age groups (40–64) | | 12 (−22 to 47) |
| Mall visit × Age groups (≥65) | | **−172 (−224 to −120)** |
| Mall visit × Maximum temperature (≤15.0°C) × Age groups (40–64) | | −15 (−51 to 22) |
| Mall visit × Maximum temperature (≤15.0°C) × Age groups (≥65) | | 14 (−41 to 69) |
| Mall visit × Maximum temperature (≥35.0°C) × Age groups (40–64) | | **−101 (−171 to −31)** |
| Mall visit × Maximum temperature (≥35.0°C) × Age groups (≥65) | | 86 (−19 to 191) |
| Mall visit × Precipitation (0–4.9 mm) × Age groups (40–64) | | −9 (−51 to 34) |
| Mall visit × Precipitation (0–4.9 mm) × Age groups (≥65) | | **128 (65 to 191)** |
| Mall visit × Precipitation (≥5.0 mm) × Age groups (40–64) | | −5 (−48 to 37) |
| Mall visit × Precipitation (≥5.0 mm) × Age groups (≥65) | | **233** **(169 to 296)** |
| Mall visit × Snow depth (0–2.9 cm) × Age groups (40–64) | | 10 (−104 to 125) |
| Mall visit × Snow depth (0–2.9 cm) × Age groups (≥65) | | −59 (−232 to 114) |
| Mall visit × Snow depth (≥3.0 cm) × Age groups (40–64) | | 34 (−92 to 161) |
| Mall visit × Snow depth (≥3.0 cm) × Age groups (≥65) | | −7 (−197 to 184) |
| Mall visit × Wind speed (1.74–2.50 m/s) × Age groups (40–64) | | −8 (−46 to 31) |
| Mall visit × Wind speed (1.74–2.50 m/s) × Age groups (≥65) | | −1 (−58 to 57) |
| Mall visit × Wind speed (>2.50 m/s) × Age groups (40–64) | | −9 (−49 to 30) |
| Mall visit × Wind speed (>2.50 m/s) × Age groups (≥65) | | 10 (−49 to 70) |

CI: confidence interval; ref: reference group. Bold text indicates <0.05.
Adjusted for gender, age groups, body mass index, marital status, employment status, education, annual equivalent income, car use, walking behavior, self–rated health, day of the week, and population density.

Supplementary Table 3b. Mixed-effect linear regression model coefficients showing an association with the three-way interaction term involving mall visits, weather, and gender, and daily step counts.

| Daily steps | | Coefficient (95% CI) |
| --- | --- | --- |
| Mall visit | |  |
|  | No | ref |
|  | Yes | **940 (914 to 966)** |
| Maximum temperature (°C) | |  |
|  | ≤15.0 | **−214 (−231 to −196)** |
|  | 15.1–34.9 | ref |
|  | ≥35.0 | **−433 (−466 to −400)** |
| Precipitation (mm) | |  |
|  | 0 | ref |
|  | 0.1–4.9 | **−267 (−287 to −247)** |
|  | ≥5.0 | **−801 (−821 to −782)** |
| Snow depth (cm) | |  |
|  | 0 | ref |
|  | 0.1–2.9 | **−87 (−140 to −34)** |
|  | ≥3.0 | −41 (−100 to 18) |
| Wind speed (m/s) | |  |
|  | <1.74 | ref |
|  | 1.74–2.50 | **−45 (−64 to −27)** |
|  | >2.50 | **−61 (−80 to −41)** |
| Gender | |  |
|  | Men | ref |
|  | Women | **−2509 (−2636 to −2382)** |
| Mall visit × Maximum temperature (≤15.0°C) | | **93 (66 to 121)** |
| Mall visit × Maximum temperature (≥35.0°C) | | −4 (−56 to 48) |
| Mall visit × Precipitation (0–4.9 mm) | | −4 (−36 to 28) |
| Mall visit × Precipitation (≥5.0 mm) | | **93 (61 to 125)** |
| Mall visit × Snow depth (0–2.9 cm) | | −15 (−104 to 75) |
| Mall visit × Snow depth (≥3.0 cm) | | **286 (191 to 382)** |
| Mall visit × Wind speed (1.74–2.50 m/s) | | 19 (−10 to 48) |
| Mall visit × Wind speed (>2.50 m/s) | | **56 (26 to 86)** |
| Maximum temperature (≤15.0°C) × Gender (women) | | **75 (54 to 95)** |
| Maximum temperature (≥35.0°C) × Gender (women) | | **50 (11 to 89)** |
| Precipitation (0–4.9 mm) × Gender (women) | | **56 (33 to 79)** |
| Precipitation (≥5.0 mm) × Gender (women) | | **294 (271 to 317)** |
| Snow depth (0–2.9 cm) × Gender (women) | | −47 (−110 to 15) |
| Snow depth (≥3.0 cm) × Gender (women) | | −44 (−112 to 24) |
| Wind speed (1.74–2.50 m/s) × Gender (women) | | **37 (16 to 59)** |
| Wind speed (>2.50 m/s) × Gender (women) | | **45 (21 to 68)** |
| Mall visit × Gender (women) | | **448 (417 to 479)** |
| Mall visit × Maximum temperature (≤15.0°C) × Gender (women) | | 9 (−24 to 42) |
| Mall visit × Maximum temperature (≥35.0°C) × Gender (women) | | **68** **(5 to 131)** |
| Mall visit × Precipitation (0–4.9 mm) × Gender (women) | | 37 (−1 to 75) |
| Mall visit × Precipitation (≥5.0 mm) × Gender (women) | | 8 (−30 to 47) |
| Mall visit × Snow depth (0–2.9 cm) × Gender (women) | | 80 (−27 to 186) |
| Mall visit × Snow depth (≥3.0 cm) × Gender (women) | | **−198** **(−312 to −83)** |
| Mall visit × Wind speed (1.74–2.50 m/s) × Gender (women) | | −16 (−50 to 19) |
| Mall visit × Wind speed (>2.50 m/s) × Gender (women) | | **−56** **(−92 to −20)** |

CI: confidence interval; ref: reference group. Bold text indicates p<0.05.
Adjusted for gender, age groups, body mass index, marital status, employment status, education, annual equivalent income, car use, walking behavior, self–rated health, day of the week, and population density.

Supplementary Table 3c. Mixed-effect linear regression model coefficients showing an association with the three-way interaction term involving mall visits, weather, and population density, and daily step counts.

| Daily steps | | Coefficient (95% CI) |
| --- | --- | --- |
| Mall visit | |  |
|  | No | ref |
|  | Yes | **1261 (1238 to 1284)** |
| Maximum temperature (°C) | |  |
|  | ≤15.0 | **−149 (−164 to −134)** |
|  | 15.1–34.9 | ref |
|  | ≥35.0 | **−437 (−463 to −411)** |
| Precipitation (mm) | |  |
|  | 0 | ref |
|  | 0–4.9 | **−222 (−239 to −205)** |
|  | ≥5.0 | **−582 (−600 to −565)** |
| Snow depth (cm) | |  |
|  | 0 | ref |
|  | 0–2.9 | −17 (−82 to 48) |
|  | ≥3.0 | 30 (−46 to 106) |
| Wind speed (m/s) | |  |
|  | <1.74 | ref |
|  | 1.74–2.50 | **−48 (−64 to −33)** |
|  | >2.50 | **−49 (−66 to −31** |
| Population density (persons per square km of inhabitable area) | |  |
|  | Tertile 1 (low: <3,190) | **−463 (−598 to −329)** |
|  | Tertile 2 (mid: 3,190–7,259) | **−384 (−508 to −260)** |
|  | Tertile 3 (high: >7,259) | ref |
| Mall visit × Maximum temperature (≤15.0°C) | | **91 (67 to 114)** |
| Mall visit × Maximum temperature (≥35°C) | | **54 (12 to 96)** |
| Mall visit × Precipitation (0–4.9 mm) | | 18 (−10 to 46) |
| Mall visit × Precipitation (≥5.0 mm) | | **68 (39 to 96)** |
| Mall visit × Snow depth (0–2.9 cm) | | −36 (−144 to 72) |
| Mall visit × Snow depth (≥3.0 cm) | | 56 (−69 to 181) |
| Mall visit × Wind speed (1.74–2.50 m/s) | | **27 (2 to 52)** |
| Mall visit × Wind speed (>2.50 m/s) | | 14 (−12 to 40) |
| Maximum temperature (≤15.0°C) × Population density (mid) | | −14 (−35 to 7) |
| Maximum temperature (≤15.0°C) × Population density (low) | | −18 (−41 to 5) |
| Maximum temperature (≥35.0°C) × Population density (mid) | | **75 (35 to 115)** |
| Maximum temperature (≥35.0°C) × Population density (low) | | **81 (35 to 127)** |
| Precipitation (0–4.9 mm) × Population density (mid) | | −6 (−30 to 19) |
| Precipitation (0–4.9 mm) × Population density (low) | | −9 (−35 to 17) |
| Precipitation (≥5.0 mm) × Population density (mid) | | −10 (−34 to 15) |
| Precipitation (≥5.0 mm) × Population density (low) | | −2 (−28 to 24) |
| Snow depth (0–2.9 cm) × Population density (mid) | | −75 (−154 to 4) |
| Snow depth (0–2.9 cm) × Population density (low) | | **−161 (−237 to −84)** |
| Snow depth (≥3.0 cm) × Population density (mid) | | −26 (−116 to 65) |
| Snow depth (≥3.0 cm) × Population density (low) | | **−182 (−269 to −94)** |
| Wind speed (1.74–2.50 m/s) × Population density (mid) | | **45 (23 to 68)** |
| Wind speed (1.74–2.50 m/s) × Population density (low) | | **50 (26 to 74)** |
| Wind speed (>2.50 m/s) × Population density (mid) | | **39 (14 to 63)** |
| Wind speed (>2.50 m/s) × Population density (low) | | 22 (−5 to 48) |
| Mall visit × Population density (mid) | | 5 (−28 to 39) |
| Mall visit × Population density (low) | | 11 (−25 to 47) |
| Mall visit × Maximum temperature (≤15.0°C) × Population density (mid) | | −11 (−46 to 24) |
| Mall visit × Maximum temperature (≤15.0°C) × Population density (low) | | 27 (−12 to 67) |
| Mall visit × Maximum temperature (≥35.0°C) × Population density (mid) | | −14 (−80 to 52) |
| Mall visit × Maximum temperature (≥35.0°C) × Population density (low) | | −49 (−127 to 29) |
| Mall visit × Precipitation (0–4.9 mm) × Population density (mid) | | −7 (−48 to 34) |
| Mall visit × Precipitation (0–4.9 mm) × Population density (low) | | 5 (−40 to 49) |
| Mall visit × Precipitation (≥5.0 mm) × Population density (mid) | | 26 (−16 to 67) |
| Mall visit × Precipitation (≥5.0 mm) × Population density (low) | | 20 (−25 to 64) |
| Mall visit × Snow depth (0–2.9 cm) × Population density (mid) | | −19 (−153 to 115) |
| Mall visit × Snow depth (0–2.9 cm) × Population density (low) | | **182** **(51 to 314)** |
| Mall visit × Snow depth (≥3.0 cm) × Population density (mid) | | −82 (−234 to 70) |
| Mall visit × Snow depth (≥3.0 cm) × Population density (low) | | **263** **(114 to 412)** |
| Mall visit × Wind speed (1.74–2.50 m/s) × Population density (mid) | | −34 (−71 to 3) |
| Mall visit × Wind speed (1.74–2.50 m/s) × Population density (low) | | −23 (−63 to 18) |
| Mall visit × Wind speed (>2.50 m/s) × Population density (mid) | | −11 (−49 to 27) |
| Mall visit × Wind speed (>2.50 m/s) × Population density (low) | | **48** **(6 to 90)** |

CI: confidence interval; ref: reference group. Bold text indicates p<0.05.
Adjusted for gender, age groups, body mass index, marital status, employment status, education, annual equivalent income, car use, walking behavior, self-rated health, day of the week, and population density.

| Supplementary Table 4. Mall visit days, all days, and the proportion of mall visits across adverse weather conditions. | | | | |
| --- | --- | --- | --- | --- |
| Weather conditions | | Mall visit days | All days | Proportion of mall visits (%) |
| Maximum temperature (°C) | |  |  |  |
|  | ≤ 15.0 | 464,077 | 1,336,129 | 34.7 |
|  | 15.1–34.9 | 1,180,686 | 3,445,886 | 34.3 |
|  | ≥ 35.0 | 93,388 | 258,064 | 36.2 |
| Precipitation (mm) | |  |  |  |
|  | 0 | 1,174,046 | 3,378,353 | 34.8 |
|  | 0–4.9 | 282,184 | 828,159 | 34.1 |
|  | ≥ 5.0 | 281,921 | 833,567 | 33.8 |
| Snow depth (cm) | |  |  |  |
|  | 0 | 1,680,467 | 4,852,892 | 34.6 |
|  | 0–2.9 | 30,760 | 97,762 | 31.5 |
|  | ≥ 3.0 | 26,924 | 89,425 | 30.1 |
| Wind speed (m/s) | |  |  |  |
|  | < 1.74 | 567,573 | 1,665,138 | 34.1 |
|  | 1.74–2.50 | 577,442 | 1,668,271 | 34.6 |
|  | > 2.50 | 593,136 | 1,706,670 | 34.8 |
| Data were derived from 18,666 participants followed for 1 year, providing a total of 5,040,079 person-days of step count data. Mall visit days and all days are expressed as person-days. A mall visit day was defined as a person-day on which the participant visited a shopping mall. All days represent the total number of person-days, including both mall visit days and non-visit days. The proportion of mall visits was expressed as the percentage of mall visit days out of the total number of all days. | | | | |

| Supplementary Table 5. Multilevel modified Poisson regression model showing associations between adverse weather conditions and mall visits. | | |
| --- | --- | --- |
| Weather conditions | | PR (95%CI) |
| Maximum temperature (°C) | |  |
|  | ≤ 15.0 | **1.02 (1.02–1.03)** |
|  | 15.1–34.9 | Ref |
|  | ≥ 35.0 | **1.01 (1.00–1.02)** |
| Precipitation (mm) | |  |
|  | 0 | ref |
|  | 0–4.9 | **0.99 (0.99–0.99)** |
|  | ≥ 5.0 | **0.99 (0.98–0.99)** |
| Snow depth (cm) | |  |
|  | 0 | ref |
|  | 0–2.9 | 1.00 (0.99–1.01) |
|  | ≥ 3.0 | **0.96 (0.95–0.98)** |
| Wind speed (m/s) | |  |
|  | < 1.74 | ref |
|  | 1.74–2.50 | **1.01 (1.00–1.01)** |
|  | > 2.50 | **1.01 (1.00–1.01)** |
| PR: prevalence ratio; CI: confidence interval; ref: reference group. Bold text indicates p < 0.05. Adjusted for gender, age groups, body mass index, marital status, employment status, education, annual equivalent income, car use, walking behavior, self–rated health, day of the week, and population density. | | |
